# Mechanism and Parameter Optimization in Grinding and Polishing of M300 Steel by an Elastic Abrasive

**DOI:** 10.3390/ma12030340

**Published:** 2019-01-22

**Authors:** Xin Tong, Xiaojun Wu, Fengyong Zhang, Guangqiang Ma, Ying Zhang, Binhua Wen, Yongtang Tian

**Affiliations:** School of Mechanical and Electrical Engineering, Xi’an University of Architecture and Technology, Xi’an 710055, China; tong24xin@163.com (X.T.); zhangfy12@126.com (F.Z.); ma15556515658@163.com (G.M.); zhangying831216@163.com (Y.Z.); wenbinhua123@163.com (B.W.); beitangwenfu@163.com (Y.T.)

**Keywords:** M300 mold steel, elastic abrasive, PSO-BP neural network algorithm, parameter optimization

## Abstract

In order to achieve high quality polishing of a M300 mold steel curved surface, an elastic abrasive is introduced in this paper and its polishing parameters are optimized so that the mirror roughness can be achieved. Based on the Preston equation and Hertz Contact Theory, the theoretical material removal rate (MRR) equation for surface polishing of elastic abrasives is obtained. The effects of process parameters on MRR are analyzed and the polishing parameters to be optimized are as follows: particle size (S), rotational speed (Wt), cutting depth (Ap) and feed speed (Vf). The Taguchi method is applied to design the orthogonal experiment with four factors and three levels. The influence degree of various factors on the roughness of the polished surface and the combination of parameters to be optimized were obtained by the signal-to-noise ratio method. The particle swarm optimization algorithm optimized with the back propagation (BP) neural network algorithm (PSO-BP) is used to optimize the polishing parameters. The results show that the rotational speed has the greatest influence on the roughness, the influence degree of abrasive particle size is greater than that of feed speed, and cutting depth has the least influence. The optimum parameters are as follows: particle size (S) = #1200, rotational speed (Wt) = 4500 rpm, cutting depth (Ap) = 0.25 mm and feed speed (Vf) = 0.8 mm/min. The roughness of the surface polishing with optimum parameters is reduced to 0.021 μm.

## 1. Introduction

Due to its high Cr (16%) content, M300 mold steel has good corrosion resistance and wear resistance and has strong resistance to the erosion of general chemicals. It is often used in molds for various kinds of plastics, such as transparent plastics, camera lenses and so on. As one of the most important processes of mold surface disposing, mold polishing directly influences the quality of the mold surface and its performance. At present, mold polishing mainly adopts traditional manual polishing, which is time-consuming and laborious, and the polishing quality is difficult to guarantee [1,2]. Although manual polishing can meet the requirements of precision mold surface finish (Ra > 0.04–0.08 μm) [3], its time-consuming and laborious shortcomings make it difficult to meet the requirements of modern industry for low cost, short cycle and high quality. 

In modern mold manufacturing, the proportions of free-form surfaces are increasing, and higher requirements of mold processing technology are required [4]. When the elastic abrasive tool is in contact with the surface of hard steel, the deformation of the surface of the abrasive tool is completely elastoplastic. The elastic abrasive can have well-profiled contact with a curved surface workpiece on account of its polymer elastic abrasive binder structure with greater flexibility, which is different from a rigid fixed abrasive grinding wheel where fretting of adjacent abrasives may happen on partial surface [5]. It is beneficial to improve the quality of curved surface polishing using an elastic abrasive. At the same time, an excellent profiling effect makes the elastic abrasive polishing suitable for free-form surfaces.

Conventional automated polishing uses free abrasive particles. This material is suitable for aspherical parts and workpiece surfaces with a small curvature. This method has low processing efficiency and high processing cost. The contact pressure between the polishing tool and the contact surface needs to be measured using a pressure sensor, since the conventional polishing tool is inelastic [6,7]. Compared with the free abrasive particle polishing process, the fixed abrasive polishing process has a large number of abrasive grains [8]. This type of material has a high material removal rate and a good self-twisting effect. The fully elastic contact characteristics allow the contact pressure to be determined by the depth of the cut. Due to the complex non-linear relationship between polishing parameters and roughness, the objective function that needs to be optimized cannot be obtained. Other optimization methods, such as particle swarm optimization or ant colony algorithm, are no longer suitable. However, the back propagation (BP) neural network has strong adaptive and self-organizing capabilities and is widely used in data prediction and numerical analysis.

The surface polishing mechanism and parameter optimization have been deeply studied. Beaucamp has studied the shape adaptive grinding process, which has been applied to finishing titanium alloy (Ti6Al4V) additively manufactured by EBM (Electron Beam Machining) and SLS (Selective Laser Sintering) [9]. However, this type of polishing method did not meet the requirements of precision mold surface finish. Zhang has studied the parameter optimization of five-axis polishing using an abrasive belt flap wheel for a blisk blade, in which RSM (Response Surface Methodology) is used to analyze the interactions of polishing factors on SR (Surface Roughness) and establish a predictive model between SR and various parameters [10]. A multi-objective particle swarm optimization algorithm (MOPSOA) is applied to optimize surface roughness of a work-piece after circular magnetic abrasive polishing by Nguyen [11]. A statistics parameters optimization method based on index atlases is presented for a novel 5-DOF (5-Degree of Freedom) gasbag polishing machine tool by Yan [12]^.^ However, for elastic abrasive polishing of M300 mold steel, there is still no complete study about parameter optimization.

In order to realize high quality polishing for a M300 mold steel curved surface, based on the Preston equation and Hertz Contact Theory, the polishing mechanism of the elastic abrasive is studied in this paper [13]. The automatic polishing experiment of M300 steel was carried out using elastic abrasive tools of varying particle size. The influence of abrasive particle size, abrasive rotational speed, cutting depth and feed speed on surface roughness was analyzed. 

Traditional BP neural networks use error back propagation to adjust the connection weight of the network. The BP neural network is quick to fall into the local optimal solution, the convergence speed is slow, and the network training is unstable. Therefore, the particle swarm optimization (PSO) algorithm is used to optimize the network weight and threshold to improve the network accuracy and convergence speed.

The BP Neural Network algorithm, which is optimized by the Particle Swarm Optimization algorithm (PSO-BP), is then used to achieve the optimal parameter combination. Finally, the surface quality, which is polished under the conditions of the optimal parameter combination, is verified by experiments.

## 2. Experiment Design

### 2.1. Experiment Devices

**Figure 1:**
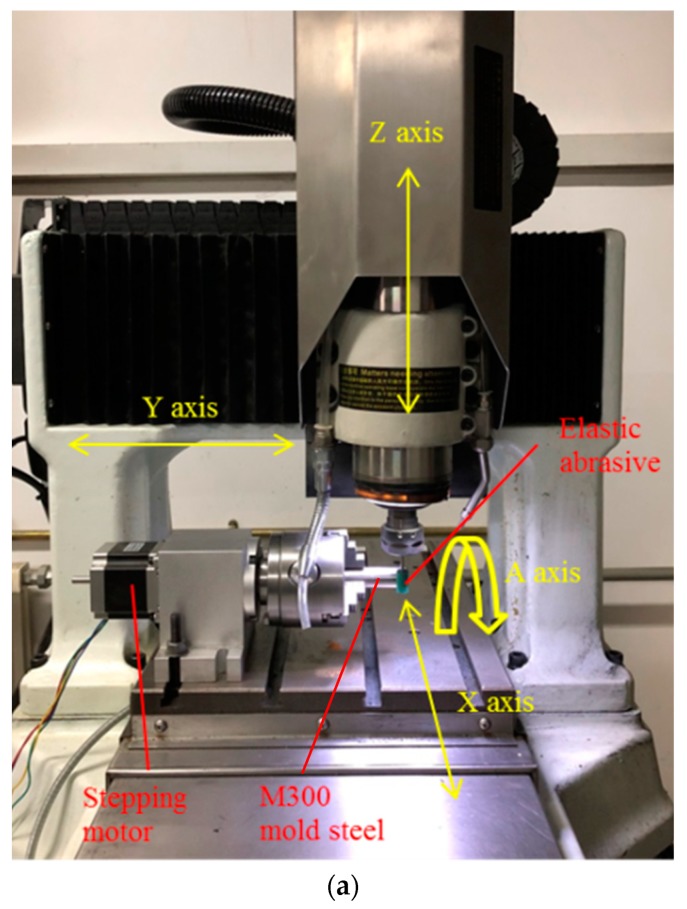

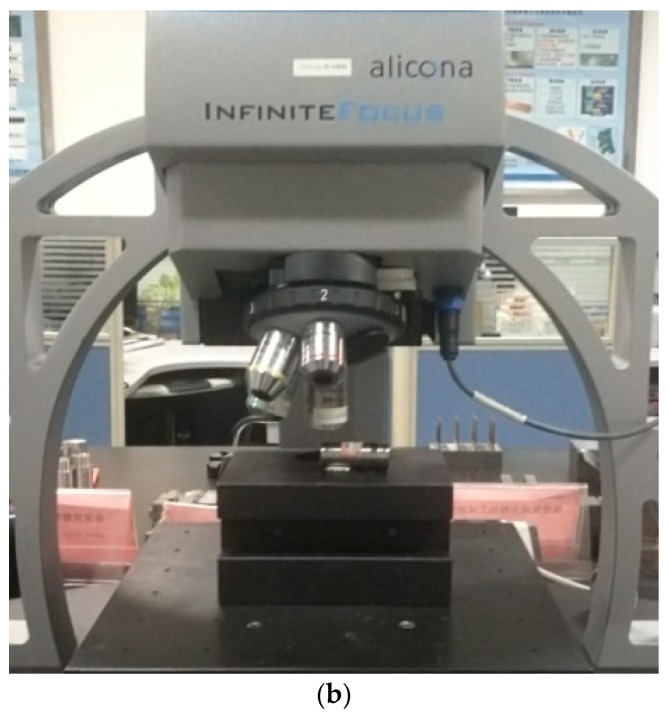
Experimental platform of polishing. (**a**) Grinding and polishing process of elastic abrasive tool. (**b**) Measurement of workpiece surface roughness.

The experiments were carried out on 4-axis precision Computer Numerical Control (CNC) machine tool Mikoni430P, which is produced by LuoYang Mikoni Precision Machinery Co., Ltd., LuoYang, China. The device includes three moving axes X, Y and Z (the repeated positioning accuracy is 1 μm), and a rotation axis, A. The polishing experimental platform is shown in Figure 1a.

The surface quality of the workpiece and abrasive is measured by an Alicona INFINITE Focus three-dimensional (3D) profilometer, as shown in Figure 1b. This equipment is produced by Alicona, Austria. The magnification and the size view of the lens, which is applied for specimen measuring, are 100×/0.6 and 285.0027 µm × 216.2089 µm. The parameter settings for abrasive measuring are 20×/0.4 and 713.7553 µm × 541.4695 µm, respectively. The high pass filter is Gaussian filtering. The experiments are carried out three times to reduce the influence of random factors. When the workpiece is rotated in the YOZ plane, the polishing trajectory is strictly center symmetrical [14]. However, the workpiece in this experiment only rotates in the XOZ plane, which causes the cutting of abrasive grains mainly in the horizontal direction. Thus, the roughness (Ra) is measured along with the axial direction of the workpiece.

### 2.2. Experiment Conditions

The specimen size is Ф18 × 55 mm and the material is M300 steel. The chemical composition of the material is shown in Table 1. The surface roughness (Ra) of the workpiece has decreased to 0.8–1 μm after preliminary semi-finishing. The workpiece is fixed on the rotation work table, which has a rotational speed of 300 r/min.

The polishing experiments are carried out using elastic abrasives of various grain size under different process parameter combinations. The experimental conditions have been presented in Table 2.

## 3. The Polishing Mechanism Using Elastic Abrasive

### 3.1. Factors of the Material Removal Rate 

The mechanism of elastic abrasive tools in the polishing process is complex. The elastic–plastic deformation of abrasive surfaces and the continuous wear of the contact area lead to the decrease and fluctuation of contact surface pressure [15]. The model of material removal for the polishing process can be established according to the Preston equation for the surface polishing by axial feed abrasive on a self-rotating workpiece. The Preston equation is a commonly used empirical formula for material removal rate, which reveals that the material removal depth by a single abrasive grain is proportional to the relative pressure and line speed of the abrasive. The material removal rate (MRR) of grain in unit length of track can be expressed by Formula (1) [16]:

(1) $$\frac{\mathrm{dh}}{\mathrm{dl}}{=K}_{p}\frac{V_{s}{+V}_{f}}{V_{f}}P$$

where Kp is the correction factor, which is related to the hardness of the workpiece and the abrasive grain, as well as the abrasive grain size; Vs is the tangential line speed of abrasive; Vf is the axial feed speed along the workpiece; and P is the pressure on the contact zone.

**Figure 2:**
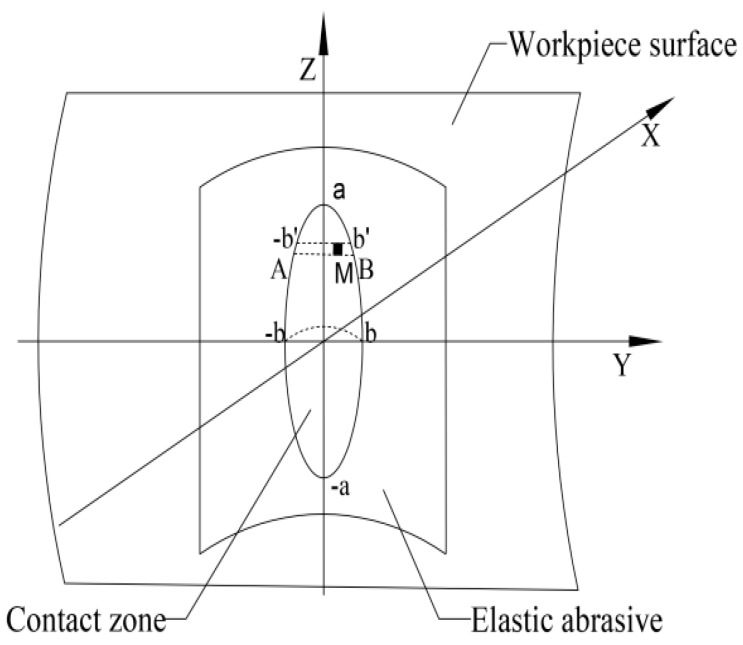
Contact institution of an elastic abrasive and the workpiece surface.

According to the Hertz Contact Theory, the polishing process can be simplified as the contact situation between the rigid body (workpiece) and the elastic body (abrasive). The contact surface between the workpiece and abrasive tool is ellipse as shown in Figure 2. The contact pressure submits to Elliptical Hertz distribution [17]:

(2) $$P\left(y,z\right)=-P_{0}\sqrt{1-{\left(\frac{z}{a}\right)}^{2}-{\left(\frac{y}{b}\right)}^{2}}$$

**Figure 3:**
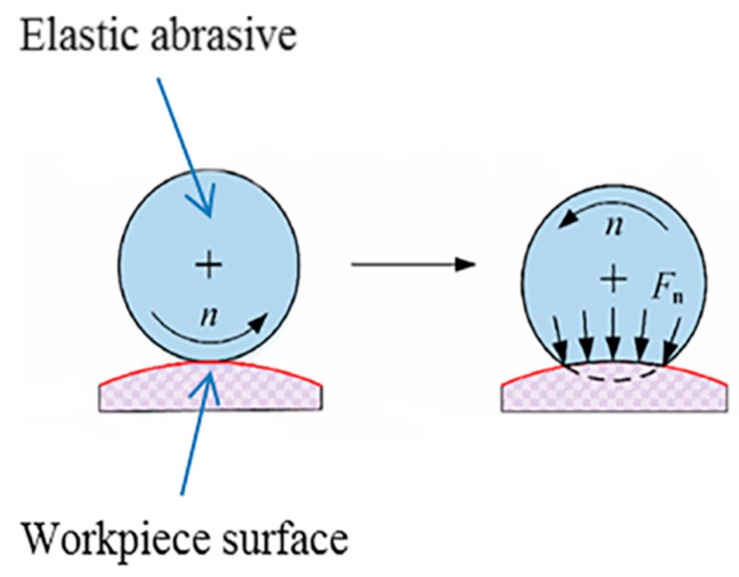
Contact force of an elastic abrasive and the workpiece surface.

${\mathrm{where}\;P}_{0}=\frac{{3F}_{n}}{2\pi \mathrm{ab}}$ is the center pressure in the contact zone and Fn is the contact force when polishing, as shown in Figure 3. The elastic abrasive tool is deformed when the workpiece is pressed. Thus, the contact force is perpendicular to the tangent of the workpiece surface.

The material removal amount of the infinitesimal M along the Y direction in contact region AB is:

(3) $$h\left(x\right)=\int_{-b^{\prime }}^{b^{\prime }}\frac{\mathrm{dh}}{\mathrm{dl}}\mathrm{dy}=\int_{-b^{\prime }}^{b^{\prime }}K_{p}P\frac{V_{s}{\pm V}_{f}}{V_{f}}\mathrm{dy}$$

in the formula b′=b1−(xc)2. The theoretical equation of MRR on the workpiece surface can be taken from Formula (2) and Formula (3):

(4) $$h\left(x\right)=-K_{p}\frac{V_{s}\pm V_{f}}{V_{f}}\frac{3F_{n}}{\pi a}\frac{x}{c}\sqrt{1-{\left(\frac{x}{c}\right)}^{2}}$$

Formula (4) shows that the MRR can be controlled by Vs, Vf and Fn. The elastic abrasive can be considered hyperelastic, and the contact pressure (Fn) of the workpiece surface is approximately proportional to the cutting depth of the abrasive tool [18]. Since the elastic abrasive steel is elastoplastically deformed when in contact with steel, the contact pressure is proportional to the cutting depth; the contact is replaced with cutting depth [19]. During the surface polishing experiment, Vs reflects the grinding tool speed Wt, Vf reflects the feed rate along the axis and Ap stands for the set cut depth of the abrasive tool. 

### 3.2. Research on Parameters Affected by MRR

The abrasive cutting process attributed to the contact characteristics of the elastic abrasive can be roughly divided into three stages: sliding, ploughing and the cutting process [20].

**Figure 4:**
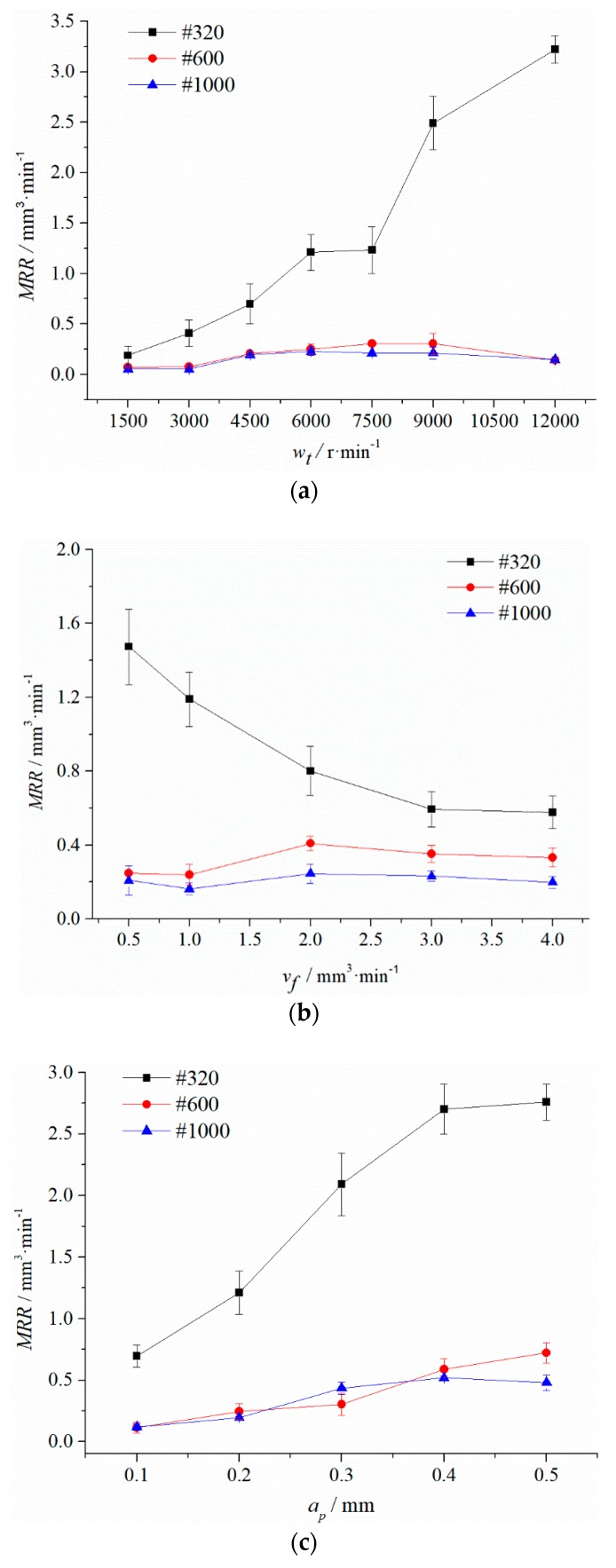
Plots of parameter effect on material removal rate (MRR) (**a**) Effect of the grinding tool speed (Wt) (**b**) effect of the feed rate (Vf) (**c**) effect of the set cut depth (Ap).

Figure 4 shows the effects of processing parameters on MRR, in which the error bars indicate the range of values after three repetitions. The MRR is approximately proportional to Wt and inversely proportional to Vf in general, which is consistent with the theoretical model.

**Figure 5:**
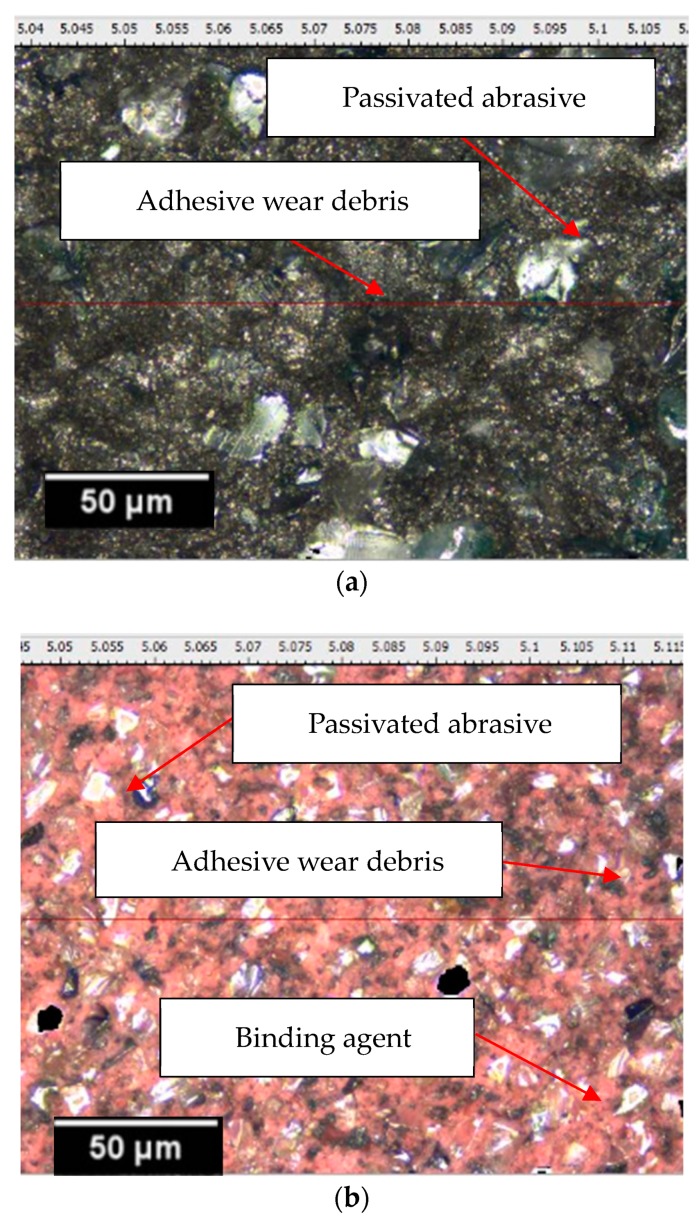

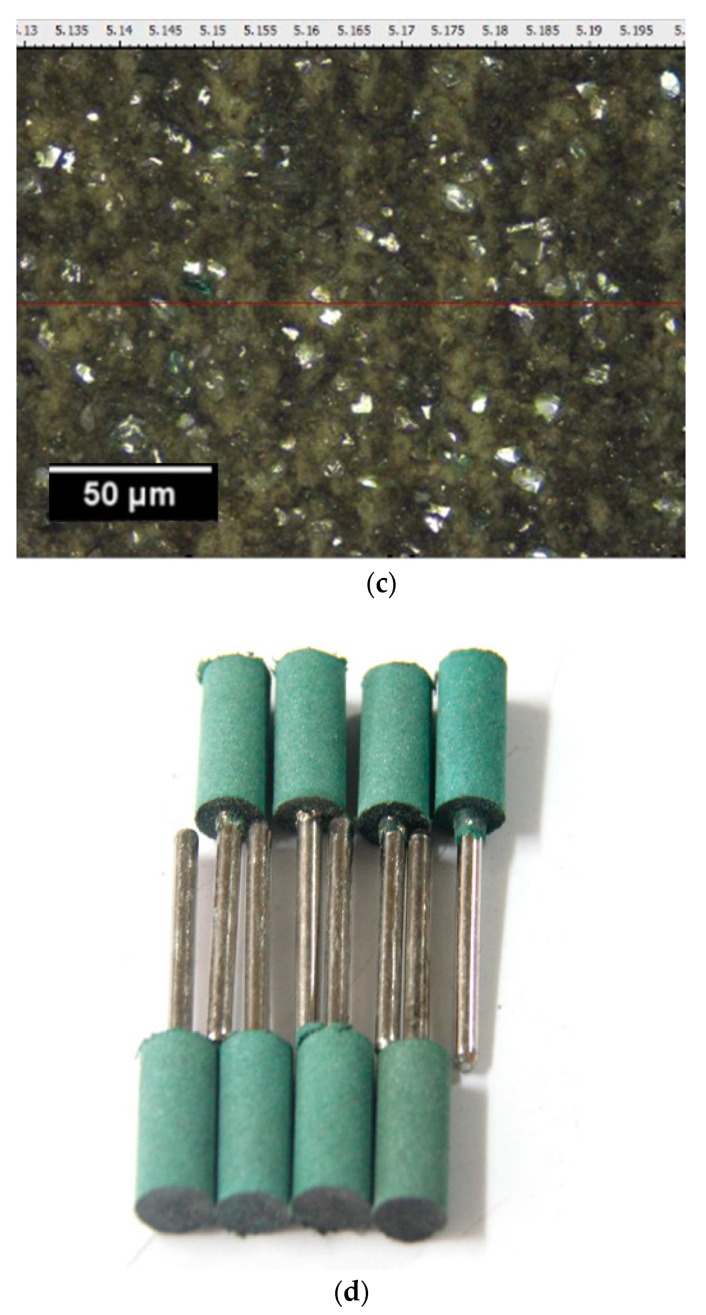
Images of all the three abrasives. (**a**) Morphological features of #320 (**b**) morphological features of #600 (**c**) morphological features of #1000 (**d**) elastic abrasive product.

The smaller the particle size, the higher the MRR. Figure 5 shows that the larger the particle size, the more the abrasive grains have in per unit area and the smaller the size of every abrasive grain. Thus, a larger particle size leads to a higher number of abrasive grains in the unit contact zone and lower average pressure on a single grain. There will be more particles in the sliding friction and ploughing processes compared with a small particle size [21], while in the stage of cutting grain, the number decreased, so the material removal capacity is low.

The appearance of the elastic grinding head after wear is shown in Figure 5. It can be seen that most of the abrasive grains have passivated facets on the top and the surface is relatively smooth. In addition, the surface of each size abrasive exhibits different degrees of adhesive wear, and the #320 abrasive is covered with more fine grinding debris than the #600 abrasive. This indicates that the smaller the particle size of the abrasive tool, the higher the removal rate of the workpiece.

In Figure 4a, the MRR of the #320 grinding tool is approximately proportional to the grinding speed (Wt), which is consistent with the material removal model, and the rate of increase is gradually slowing down at 9000 r/min. The MRR of #600 and #1000 reached the peak at 6000 r/min and 9000 r/min, respectively. Then the MRR begins to decrease. Due to the contact time between the workpiece and the larger abrasive being too short with the increase of Wt and the sliding and ploughing effect being more important than the cutting effect, the removal ability of the abrasive decreased.

In Figure 4b, for a fixed cutting depth and speed of the grinding tool, the increase of Vf increases the line spacing between the polishing tracks, and the number of passes of the grinding tool decreases per unit length. Thus, the residual height of the workpiece surface increases and the material removal rate decreases [20]. After Vf is greater than 2 mm/min, the MRR gradually decreases to a stable value.

In Figure 4c, the total MRR is approximately proportional to the set depth (Ap). By the elastic contact theory, the contact area increases with the increase of Ap. The increase in the number of abrasive particles involved in grinding and polishing increases the MRR. After Ap is greater than 0.4 mm, the MRR increase of #320 and #1000 grinding tools slows down. While the #600 grinding tool reaches the peak and then decreases when the Ap is 0.4 mm, the critical value of the depth of cut is estimated to be 0.4–0.5 mm. When the Ap is larger than the critical value, the change of the effective working area is small. At the same time, the increase of contact pressure makes the abrasive blunt off worse. Eventually, the upward trend of material removal rate slows down or even declines.

## 4. Parameter Optimization 

### 4.1. Experimental Design and Results 

Taking into account the interaction among the factors, an orthogonal experiment with four factors and three levels [22] was designed based on the Taguchi method [23], which is shown in Table 3. The processing time of each group of experiments is 180 s. In order to reduce the processing error, each group of experiments is processed three times. The result is taken as its average value, which is as shown in Table 4.

In the table above: Mean 1 is the mean of the normal variance of the surface roughness of the influencing factor in the level 1 combination.

Mean 2 is the mean of the normal variance of the surface roughness of the influencing factor in the level 2 combination.

Mean 3 is the mean of the normal variance of the surface roughness of the influencing factor in the level 3 combination.

The combination of grinding parameters for a single optimized target can be achieved by the signal-to-noise ratio (SNR) analysis of the experimental data. Since the optimization target is surface roughness (Ra), the design parameters of small characters are adopted, such as Formula (5)

(5) $$\mathrm{SNR}=-10\mathrm{lg}\overset{n}{\underset{i=1}{\sum }}R_{i}^{2}$$

Table 5 is the average response of SNR to Ra in each parameter level. The larger the SNR, the higher the parameter influence on Ra will be. It can be seen that the abrasive grain size and the abrasive speed have a high influence on Ra.

In order to express the influence trend of each factor level on the surface roughness more intuitively, the main effect diagram of polished roughness is obtained. 

When the particle size is too small, the residual peak on the surface of the workpiece is not sufficiently cut, so that when the particle size increases, the roughness decreases. When the grinding speed is too fast, it results in incomplete cutting. However, if the speed is too slow, it will result in a decrease in the number of abrasive grains involved in cutting per unit time. When the depth of the cut increases, the roughness decreases due to over-cutting of the abrasive grains. As the depth of cut further increases, the deformation of the abrasive increases and the contact area with the workpiece increases. Finally, the time of cutting of the abrasive is increased, and the roughness decreases. Excessive feed rates and low feed rates will result in undercutting and overcutting, respectively.

**Figure 6:**
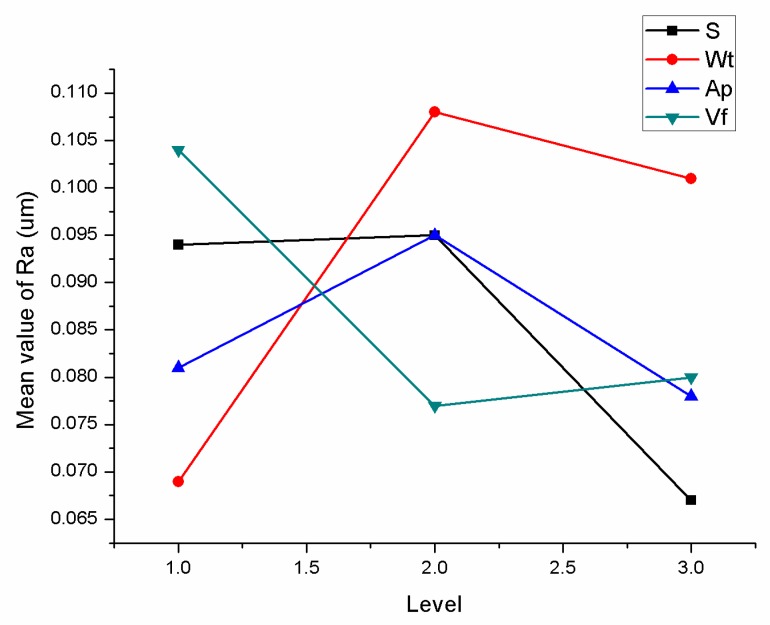
Main effect diagram of Ra.

As shown in Figure 6, particle size (S), grinding speed (Wt), cutting depth (Ap) and feed velocity (Vf) are at minimum roughness at levels 3, 1, 3, 2, respectively. The minRa parameter combinations are A3 B1 C3 D2, since the roughness is negative indicator.

### 4.2. PSO-BP Neural Network Model

The surface roughness polished with an elastic abrasive is affected by many factors, and the complex non-linear relationship between roughness and influencing factors is difficult to fit to a linear model or common non-linear model. The BP neural network has a high mapping ability and can realize any non-linear mapping from input to output. By using this high mapping ability and generalization ability of the BP neural network, the mapping model between particle size (S), rotational speed (Wt), cutting depth (Ap), feed speed (Vf) and polished surface roughness can be established to solve the problem of parameter optimization. However, the BP neural network easily falls into the local extremum [24].

Particle swarm optimization (PSO) is a swarm intelligence optimization algorithm which finds out the optimal region in a complex search space by the interactions among particles [25]. The learning of the BP neural network is mainly reflected in the adjustment process of the weight value and the threshold. The optimization operation of particle swarm optimization corresponds to the weight value and the threshold of the BP neural network algorithm, and then the PSO-BP neural network model is established.

**Figure 7:**
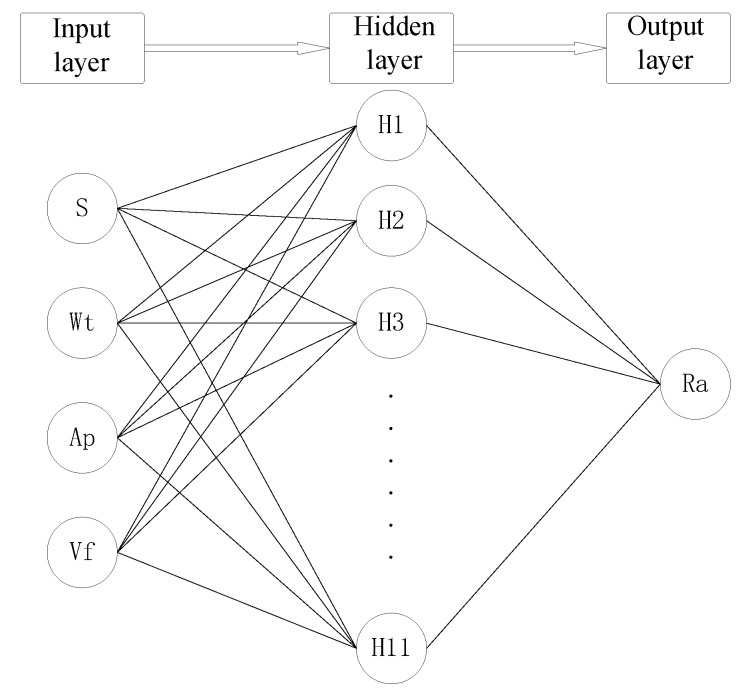
Schematic diagram of back propagation (BP) neural network structure.

Particle size (S), rotational speed (Wt), cutting depth (Ap) and feed speed (Vf) are input factors. The polishing surface roughness is used as the output factor. The BP neural network model with one hidden layer is established, as shown in Figure 7. The number of neurons in the hidden layer is 11. The transfer function of the hidden layer is “tansig”. The transfer function of the output layer is “pureline”. The training function is “trainlm”. The training accuracy, learning rate and cycle times are 0.0001, 0.05 and 3000, respectively. 

When the weight is optimized by particle swarm optimization, the connection weights of each layer of the neural network are encoded into particles and the fitness is the mean square error of the network output. The goal is to search for the optimal network weights within the default number of iterations.

The PSO algorithm functions to find the optimal solution in a group of particles by iterating. The particle is updated by the Pbest values and the Gbest values. The Pbest is the best location which is searched by particles. The Gbest is the best location which is searched by the whole particle swarm.

Supposing z_i_ = (z_i1_,z_i2_,…,z_id_,…z_iD_) is the D-dimensional position vector of the No.i particle, the position of the particle can be measured by the fitness function. The v_i_ = (v_i1_,v_i2_,…,v_id_,…,v_iD_) is the fly velocity of particle i. The p_i_ = (p_i1_,p_i2_,…,p_id_,…,p_iD_) is the optimal position of particle i so far. The p_g_ = (p_g1_,p_g2_,…,p_gd_,…,p_gD_) is the optimal position found so far by the particle swarm. The fly velocity and position are updated according to Formula (6).

(6) $$v_{\mathrm{id}}^{k+1}{=\mathrm{wv}}_{\mathrm{id}}^{k}{+c}_{1}r_{1}\left(p_{\mathrm{id}}-z_{\mathrm{id}}^{k}\right){+c}_{2}r_{2}\left(p_{\mathrm{gd}}-z_{\mathrm{id}}^{k}\right)\;i=1,\;2,\ldots ,\;m\;d=1,\;2,\ldots ,\;D$$

where k is the current number of iterations; r_1_, r_2_ is the random number (0, 1); c_1_, c_2_ are the learning factors; and W is inertia weight.

In order to maintain the equilibrium of particle swarm convergence speed and convergence efficiency, the initial algorithm should have a large global search capability and the latter algorithm should have strong local search capability. Therefore, the linear variations of Formulas (7)–(9) are used to improve the global optimization ability of the particle group at the initial stage and improve the local optimization ability of the particle group in the later stage.

(7) $$c_{1}=\left(c_{1f}\;-{\;c}_{1i}\right)\times \left({k÷k}_{\max}\right){+c}_{1i}$$

(8) $$c_{2}=\left(c_{2f}\;-{\;c}_{2i}\right)\times \left({k÷k}_{\max}\right){+c}_{2i}$$

(9) $${w=w}_{\max}-\frac{w_{\max}-w_{\min}}{k_{\max}}\times k$$

In general, when C_1_ + C_2_ < 4, the optimization ability of the example group is best [16], so c_1f_ and c_1i_ are 0.5 and 2.5, respectively; c_2f_ and c_2i_ are 2.5 and 0.5, respectively. wmax and wmin are 0.9 and 0.4, respectively.

**Figure 8:**
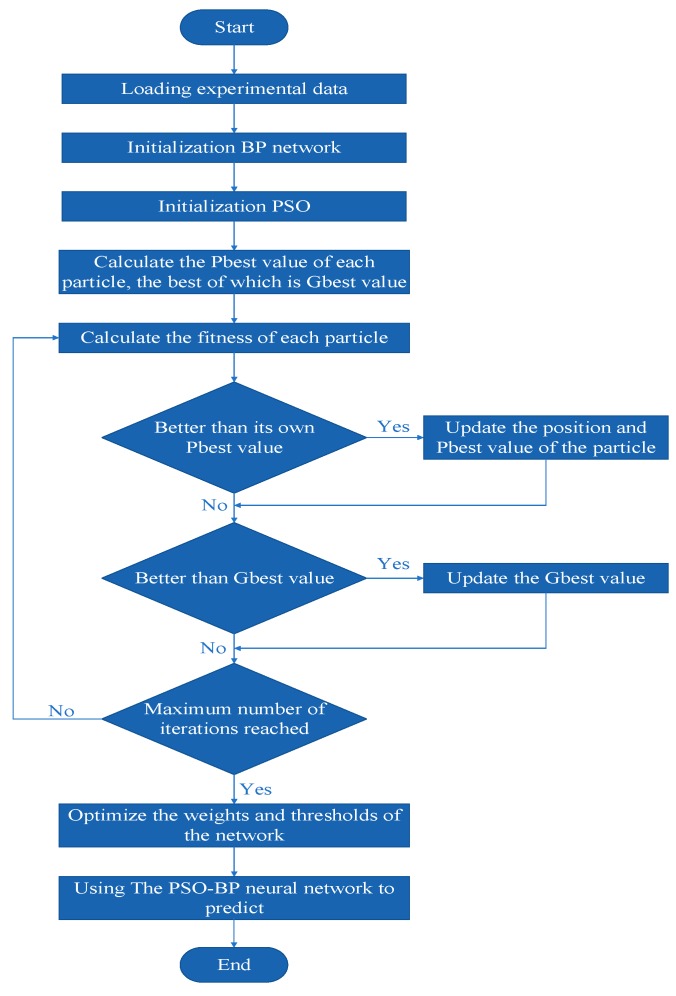
Flow-process diagram of the particle swarm optimization algorithm optimized with the back propagation neural network algorithm (PSO-BP).

Setting the maximum speed as 0.8, the number of particles as 40, and the minimum error as 0.001, a PSO-BP network model was built (Figure 7) to train the data for rows 1–25 in Table 4. The data from rows 26–30 is used to examine the trained network model. The comparison between the PSO-BP neural network and the BP neural network is shown in Figure 8.

**Figure 9:**
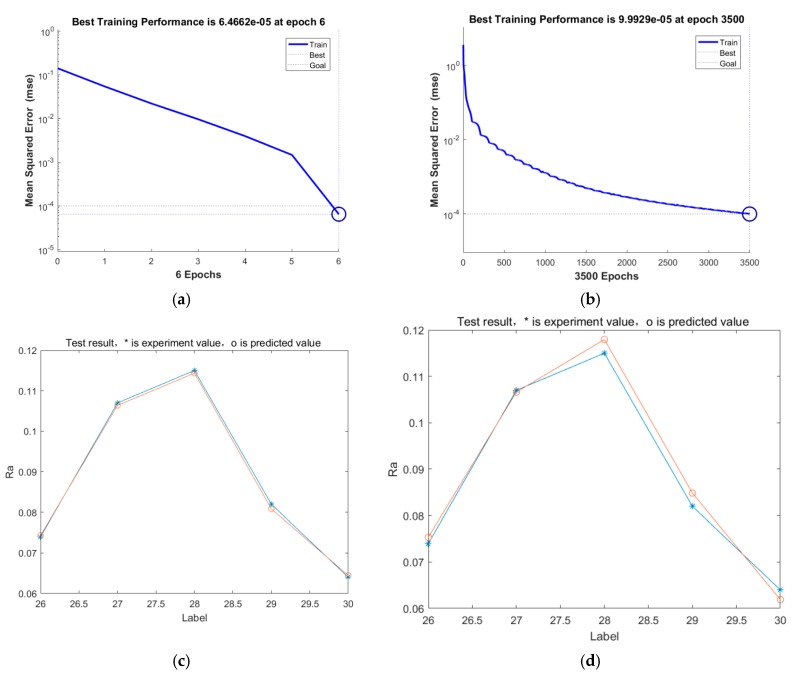
The comparison of PSO-BP and BP: (**a**) Training process of the PSO-BP neural network algorithm; (**b**) training process of the basic BP neural network algorithm; (**c**) Prediction error of the PSO-BP neural network algorithm; (**d**) Prediction error of the basic BP neural network algorithm.

Compared with Figure 9a and Figure 9b,e, it can be seen that the PSO-BP neural network converges to the preset precision in only six steps, and the efficiency of the PSO-BP neural network is obviously improved compared with the basic BP neural network. By comparing Figure 9c with Figure 9d, the predicted value of the former is very close to the experimental value, but the latter has a large deviation.

As shown in Table 6, the prediction error of the PSO-BP network model is within 0.3%, so the PSO-BP network model has a high accuracy and can be used as a prediction model.

### 4.3. Optimization Results

Based on the minRa parameter combination of each factor, each factor is set to be five levels, and the distribution is shown in Table 7. The orthogonal test is designed by using the Taguchi method and the data is input into the trained PSO-BP neural network model for prediction. The results are shown in Table 8. 

### 4.4. Experimental Verification

In Table 8, the optimized polishing parameter combination is obtained as follows: A5 B3 C2 D1 (S: #1200, Wt: 4500 rpm, Ap: 0.25 mm, Vf: 0.8 mm/min). The confirmatory experiments of the minRa parameter combination A3 B1 C3 D2 and optimized parameter combination A5 B3 C2 D1 are carried out respectively. 

**Figure 10:**
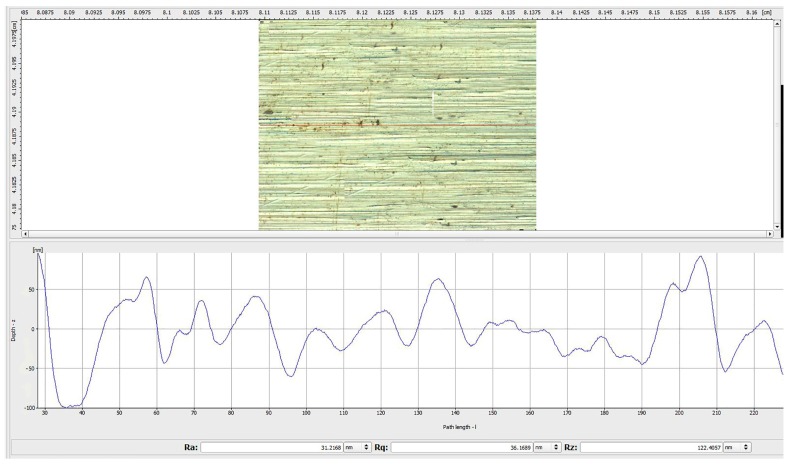
Surface topographies of the workpiece polished by optimized parameters.

**Figure 11:**
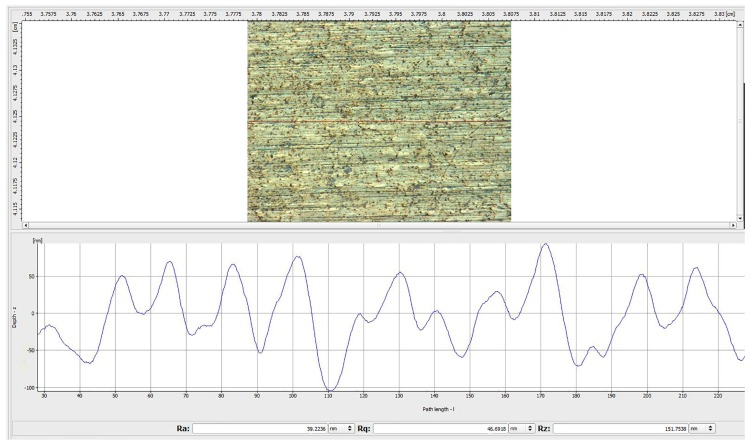
Surface topographies of the workpiece polished by the minRa parameter combination (S = #1000, Wt = 4500 r/min, Ap = 0.3 mm, Vf = 1 mm/min).

The surface morphology of the M300 workpiece polished under the conditions of the optimized parameter combination A5 B3 C2 D1 is shown in Figure 10. It can be seen that the polishing pattern is obviously reduced, and the surface damage is greatly improved. The surface roughness (Ra) is reduced to 0.021 μm after machining. Compared with the minRa parameter combination (as shown in Figure 11), the roughness is reduced significantly, and the surface quality is improved, which means that the parameter optimization method used is feasible.

## 5. Conclusions

The silicon carbide abrasive and silicone–rubber based elastic abrasive is cheaper and has a better profile when polishing the curved surface of M300 mold steel. It is easy to obtain high surface quality and provide a feasible method for high efficiency and high-quality polishing of M300 mold steel.

1. The elastic abrasive has high material removal ability in the initial stage of processing. With the increase in processing time, the material removal rate decreases rapidly and tends to be stable. Generally, the abrasive with large particle size (S) has low removal ability, and it is easy to obtain a stable polished surface quality. This is because the larger the particle size (S), the more the abrasive grains in per unit area and the smaller the size of every abrasive grain. A larger particle size (S) leads to a higher number of abrasive grains in the unit contact zone and a lower average pressure on a single grain.
2. Based on the parameter combinations of particle size, grinding speed, cutting depth and feed speed, an orthogonal experiment is carried out and the range analysis of the experimental results is performed. The results show that the speed of the grinding tool has the greatest influence on roughness, and the influence of particle size and feed speed on roughness is close. The degree of cutting depth is the least influential. The minRa parameters of each level are as follows: S = #1000, Wt = 4500 rpm, Ap = 0.3 mm and Vf = 1 mm/min.
3. The experimental parameters are trained and examined by the PSO-BP neural network algorithm. The results show that the prediction roughness error is less than 0.3%, which means that the network structure has high precision.
4. The surface roughness is taken as the optimization index. Based on the combination of minRa parameters, the polishing parameters are optimized by using the trained PSO-BP neural network structure. The optimization results show that the optimal parameter combination is S = #1200, Wt = 4500 rpm, Ap = 0.25 mm and Vf = 0.8 mm/min. The verified experiment shows that the roughness of the polished surface is reduced to 0.021 μm under the optimal parameter combination conditions, which is consistent with the predicted optimization results. The parameter optimization method based on the PSO-BP neural network algorithm is feasible to optimize the polishing parameters.

## Figures and Tables

**Table 1:** Chemical composition of M300 steel (%).

Name	C	Cr	Mo	Mn	Si
Content	0.38	16.00	1.00	0.4	0.40

**Table 2:** Experimental conditions for grinding and polishing.

Name	Conditions
Specification of abrasive tools	Ф10 mm silicone rubber based elastic abrasives
Abrasive and particle size	Silicon carbide (carborundum), #320 (42 μm), #600 (23 μm), #1000 (13 μm)
Cooling-down methods	Dry polishing

**Table 3:** Experimental conditions for grinding and polishing.

Processing Parameters	Level		
	1	2	3
Particle size, S (#)	320	600	1000
Abrasive tool speed, Wt (r/min)	4500	6000	7500
Setting cut depth, Ap (mm)	0.1	0.2	0.3
Feed rate, Vf (mm/min)	0.5	1	2

**Table 4:** Experimental conditions for grinding and polishing. Ra = surface roughness.

Number	S (A)	Wt (B)	Ap (C)	Vf (D)	Ra
1	320	4500	0.1	0.5	0.037
2	320	4500	0.2	1	0.074
3	320	4500	0.3	2	0.088
4	320	6000	0.3	0.5	0.069
5	320	7500	0.3	2	0.030
6	600	4500	0.1	1	0.079
7	600	4500	0.2	2	0.059
8	600	7500	0.3	1	0.072
9	600	4500	0.2	2	0.055
10	600	4500	0.3	0.5	0.108
11	600	6000	0.1	2	0.117
12	600	6000	0.2	0.5	0.145
13	600	7500	0.2	1	0.139
14	600	7500	0.3	2	0.106
15	1000	4500	0.2	0.5	0.037
16	1000	4500	0.3	2	0.047
17	1000	4500	0.1	0.5	0.072
18	1000	4500	0.2	1	0.046
19	1000	7500	0.2	1	0.035
20	1000	7500	0.1	1	0.045
21	1000	7500	0.2	2	0.111
22	1000	7500	0.3	0.5	0.105
23	320	7500	0.2	0.5	0.294
24	600	4500	0.1	1	0.083
25	600	6000	0.3	1	0.094
26	600	7500	0.1	0.5	0.074
27	1000	4500	0.1	2	0.107
28	600	6000	0.1	1	0.115
29	600	4500	0.3	1	0.082
30	320	4500	0.3	1	0.064
Mean 1	0.094	0.069	0.081	0.104	
Mean 2	0.095	0.108	0.095	0.077	
Mean 3	0.067	0.101	0.078	0.080	

**Table 5:** Signal-to-noise ratio (SNR) (dB) to surface roughness.

Parameter		S	Wt	Ap	Vf
Level	1	22.94	23.66	22.40	21.36
	2	20.81	19.59	22.05	22.83
	3	24.33	21.79	22.70	22.73

**Table 6:** Predicted error.

Number	26	27	28	29	30
Actual value (μm)	0.074	0.107	0.115	0.082	0.064
Predicted value (μm)	0.0741	0.1067	0.1148	0.0818	0.0643
Error(%)	0.14	0.28	0.17	0.24	0.47

**Table 7:** The distribution of each factor.

Processing Parameters	Level				
	1	2	3	4	5
Particle size, S (#)	700	800	1000	1100	1200
Abrasive tool speed, Wt (r/min)	4300	4400	4500	4600	4700
Set cut depth, Ap (mm)	0.2	0.25	0.3	0.35	0.4
Feed rate, Vf (mm/min)	0.8	0.9	1	1.1	1.2

**Table 8:** Predicted results.

Number	A (#)	B (r/min)	C (mm)	D (mm/min)	Ra (μm)
1	700	4300	0.2	0.8	0.0850
2	700	4400	0.25	0.9	0.0971
3	700	4500	0.3	1	0.0761
4	700	4600	0.35	1.1	0.0637
5	700	4700	0.4	1.2	0.0891
6	800	4300	0.25	1	0.0896
7	800	4400	0.3	1.1	0.0702
8	800	4500	0.35	1.2	0.0705
9	800	4600	0.4	0.8	0.0621
10	800	4700	0.2	0.9	0.0611
11	1000	4300	0.3	1.2	0.0491
12	1000	4400	0.35	0.8	0.0600
13	1000	4500	0.4	0.9	0.0729
14	1000	4600	0.2	1	0.0456
15	1000	4700	0.25	1.1	0.0438
16	1100	4300	0.35	0.9	0.0548
17	1100	4400	0.4	1	0.0634
18	1100	4500	0.2	1.1	0.0393
19	1100	4600	0.25	1.2	0.0360
20	1100	4700	0.3	0.8	0.0414
21	1200	4300	0.4	1.1	0.0460
22	1200	4400	0.2	1.2	0.0339
23	1200	4500	0.25	0.8	0.0211
24	1200	4600	0.3	0.9	0.0392
25	1200	4700	0.35	1	0.0536
